# Comparative Evaluation of Qualitative and Nutraceutical Parameters in Fresh Fruit and Processed Products of ‘Lady Cot’ and Vesuvian ‘Pellecchiella’ Apricot Cultivars

**DOI:** 10.3390/foods14060945

**Published:** 2025-03-10

**Authors:** Aniello Falciano, Aurora Cirillo, Mariachiara Ramondini, Prospero Di Pierro, Claudio Di Vaio

**Affiliations:** 1 Department of Agricultural Sciences, University of Naples Federico II, Via Università 100, 80055 Portici, Italy; aniello.falciano@unina.it (A.F.); mcramondini@hotmail.it (M.R.); claudio.divaio@unina.it (C.D.V.); 2 Centre for Food Innovation and Development in the Food Industry, University of Naples Federico II, 80055 Portici, Italy

**Keywords:** *Prunus armeniaca* L., processing stability, bioactive compounds, antioxidant activity, functional food

## Abstract

Apricot cultivation plays a significant role in Italy’s agricultural landscape, with the country hosting a wide variety of traditional and international cultivars, and their cultivation, processing and transformation offer a wide margin for market expansion. Jam preparation is an ideal method to preserve apricots, and understanding their functional properties is crucial for achieving high-quality products. Vesuvian autochthonous cultivars, in particular, stand out for their unique organoleptic and nutraceutical traits, which are closely linked to the region’s pedo-climatic conditions. This study investigated two apricot cultivars, the Vesuvian ‘Pellecchiella’ and the international ‘Lady Cot’, to assess their physicochemical properties and evaluate the variation in bioactive components during the transformation process from fresh fruit to puree and jam. The two cultivars exhibited distinct phenotypic differences. The ‘Lady Cot’ produced larger fruits (61.04 g vs. 45.68 g for the ‘Pellecchiella’) with a redder epicarp coloration, making it more visually appealing for commercial purposes. Conversely, the ‘Pellecchiella’ showed higher total soluble solids (TSS) and lower titratable acidity (TA), resulting in a sweeter flavor profile that may be preferred by consumers. Specifically, the ‘Pellecchiella’ exhibited a significantly higher polyphenol content, with catechin and epicatechin levels higher by 338% and 167%, respectively. The study further analyzed the variation in nutraceutical components in the puree and jam (carotenoids, total polyphenols, and antioxidant activity by ABTS, DPPH and FRAP), throughout the processing stages. Both cultivars showed a reduction in these parameters during the transformation process. For instance, the total polyphenol content exhibited a similar reduction of approximately 61% in both cultivars. However, the ‘Pellecchiella’ retained higher values in the jam, reflecting its naturally higher initial levels in the fresh fruit, and showed higher Redness Index. Overall, the results highlight ‘Pellecchiella’ as a cultivar having superior nutraceutical properties and good bioactive compound retention during processing, making it a valuable choice for both fresh consumption and processed products. These findings have significant implications for the functional food sector, as they underscore the importance of cultivar selection and processing strategies to preserve valuable bioactive compounds. By leveraging the natural advantages of local cultivars like ‘Pellecchiella’, producers could develop premium jams or puree-based functional products aimed at health-conscious consumers.

## 1. Introduction

The fruiting apricot belongs to the species *Prunus armeniaca* L. and is typical of temperate climates, having significant economic importance. Its global production reached approximately 4 million tonnes in 2020, highlighting its relevance in both the agricultural and industrial sectors. Italy stands as the leading producer of apricots in Europe, contributing around 275,000 tonnes (https://www.fao.org/faostat/en/#data/QCL, accessed on 10 November 2021) to the global total intended both for fresh consumption and for producing processed products such as juices, purees and jams, given that it is a climacteric fruit and its shelf-life is very short [1]. The apricot germplasm diversity in Italy is notable, exhibiting variations in fruit size, shape, color, firmness, flavor, ripening time and nutritional quality [2]. Since the early 2000s, the introduction of new cultivars has allowed for an extended market supply that lasts until late August. These cultivars feature innovative genetic traits such as red skin, slow-softening flesh, Sharka (*Plum pox virus*) resistance, larger size, firmness and late ripening, which provide various advantages, but sometimes at the expense of fruit quality [3,4]. The most significant area for apricot cultivation in Italy is around Mount Vesuvius in the Campania region, accounting for about 40% of the national production. Vesuvian apricots, known for their exceptional quality, are particularly sought after for processing and use in traditional confectionery and other processed products. Recent studies confirm the suitability of these apricots for juices and syrups due to their high sugar content, while their characteristics (color, texture and flavor) make them ideal for sweets and pastries. Apricots in Campania were first described by the Neapolitan scientist Gian Battista Della Porta in 1583 in his book Suae Villae Pomarium, where he distinguished two types: ‘bericocche’ and ‘crisomele’, now known as ‘crisommole’ in Neapolitan dialect. Today, more than 70 ecotypes of apricots are recognized, grouped under the name ‘Vesuvius apricot’, cultivated on volcanic soils rich in potassium, a key element that enhances their distinctive flavor. Varieties such as ‘Boccuccia’, ‘Pellecchiella’, ‘Vitillo’, ‘Ceccona’, ‘Palummella’, ‘San Castrese’, ‘Monaco Bello’ and ‘Portici’ reflect centuries of careful selection by local farmers [5,6,7,8]. These apricots, cultivated on the slopes of Mount Vesuvius in specific municipalities within the province of Naples, typically reach maturity predominantly in the second half of June and are primarily marketed. Unlike other national cultivars, Vesuvian apricots are distinguished by their superior fruit quality, characterized by a well-balanced mix of sugars and acids and a strong aroma, which are key indicators of their premium status [9].

This regional distinction underscores the localized nature of apricot cultivation in Italy and reflects a broader diversity within the national context. Despite their lower quality, imported apricots dominate the industrial market due to their greater availability. However, recent programs aim to enhance and protect Vesuvian apricots through biochemical and nutritional characterization, strengthening their connection to the territory and promoting their identity, as well as including them in genetic improvement programs.

Additionally, these apricots are rich in polyphenols, which are bioactive compounds known for their significant health benefits, including their role in reducing oxidative stress, improving cardiovascular health and potentially lowering the risk of chronic diseases. This high polyphenol content further enhances the nutritional and functional value of Vesuvian apricots, emphasizing their importance as a valuable component of a healthy diet [8].

Bioactive compounds in apricots, such as polyphenols and carotenoids, are recognized for their antioxidant properties and health-promoting effects. However, several studies highlight their high susceptibility to thermal degradation, which can compromise the nutritional quality of processed products. Specifically, temperatures above 80 °C can accelerate oxidation and isomerization of carotenoids, resulting in a 20–40% reduction in their content [10]. Similarly, polyphenols often undergo oxidation and polymerization, leading to losses of up to 40% and a 35% decrease in antioxidant activity [11]. The extent of these changes depends on the compound type, the food matrix and the specific processing conditions [12]. Understanding how thermal treatment affects different cultivars is thus vital to optimize production processes and preserve the bioactive potential of apricot-derived products.

The aim of this study was to compare two apricot cultivars, ‘Pellecchiella’ and ‘Lady Cot’, both in their fresh form and as processed products, focusing on their distinct physicochemical and nutraceutical characteristics. ‘Lady Cot’ was selected due to its international relevance and commercial appeal, characterized by larger fruit size, redder epicarp coloration and a balanced sweet-tart flavor profile. This cultivar, originally selected in France, is known for its firmness and suitability for both fresh consumption and processing, providing a valuable comparison with the traditional Vesuvian ‘Pellecchiella’ known for its superior nutraceutical properties and unique organoleptic qualities.

The analysis focused on the fruits’ physicochemical properties, nutraceutical content (including polyphenols and carotenoids) and antioxidant activity. Additionally, the study examined the nutraceutical qualities of puree and jam to assess the content of bioactive compounds after processing. By clarifying the comparative advantages of a native Vesuvian cultivar over a widely cultivated international one, this work also provides insights for producers and the functional food industry on how to optimize processing conditions to preserve the health-related attributes of apricot-based products.

## 2. Materials and Methods

### 2.1. Plant Materials

The experimental trial was conducted on a farm located at the base of Mount Vesuvius, involving two distinct cultivars: ‘Pellecchiella’ (a native cultivar from the Vesuvian area) and ‘Lady Cot’ (a cultivar selected in France). In Table 1, all the information related to various characteristics of the fruit is provided. These include the origin, which describes the geographical and morphological characteristics of the cultivar. The table also includes information on the skin color, referring to the external appearance, and the flesh color, which describes the internal pigmentation. The table provides additional information on characteristics such as texture, which indicates the tactile quality of the fruit, and main use, describing its primary purpose, whether for fresh consumption, processing or culinary applications.

The research activities were carried out in 2023, with sample collection performed once all fruits had reached commercial maturity. Specifically, the ‘Pellecchiella’ fruits were harvested on June 27th, while the ‘Lady Cot’ fruits were collected on July 15th. Samples were taken from 10 trees per cultivar, resulting in a total of 20 kg, which was subsequently divided for various physico-chemical, nutraceutical and processing analyses. The trees were selected based on uniform vigor, were of the same age (8 years), and were grown using an open-vase training system having a planting layout of 5 × 4 m. The ‘Pellecchiella’ and ‘Lady Cot’ trees were grafted onto Myrobalan 29 C rootstock. The orchard received standard horticultural maintenance, and pest-control measures were implemented in accordance with regulations governing integrated production practices.

### 2.2. Reagents

HPLC grade methanol, ethanol, hexane, formic acid and acetonitrile were sourced from Merck (Darmstadt, Germany). Analytical standards including gallic acid, β-carotene, Trolox ((±)-6-hydroxy-2,5,7,8-tetramethyl-chromane-2-carboxylic acid), ABTS (2,2-azinobis(3-ethylbenzothiazoline-6-sulphonic acid) diammonium salt), ferrous chloride, DPPH (1,1-diphenyl-2-picrylhydrazyl), TPTZ (2,4,6-tris(2-pyridyl)-1,3,5-triazine) and Folin–Ciocalteu reagent were obtained from Sigma Aldrich (Milan, Italy). All additional chemicals used were of analytical quality.

### 2.3. Physico-Chemical Characterization of Fruits

The determination of the physico-chemical parameters of the fruits was carried out on a sample consisting of 15 apricot fruits picked at the stage of consumer ripeness, which demonstrated notably low pulp firmness. Indeed, the softening of apricots is a critical factor throughout the entire post-harvest distribution chain, significantly influenced by the type of cultivar, the storage conditions and the length of storage [13]. The parameters in this study included fruit weight (g) measured using an electronic digital scale (Precisa Instruments AG, model XB220A, Dietikon, Switzerland), polar diameter (mm), equatorial diameter (mm), transversal diameter (mm) measured using a digital vernier caliper (Mitutoyo, Kawasaki, Japan) and pulp firmness (kg/0.5 cm^2^) measured using an EFFEGI manual penetrometer equipped with a 0.5 cm^2^ tip applied to two opposite sides of the fruit.

The total soluble solids (TSS) content, indicated in Brix°, was assessed using a digital refractometer (Atago, model PR-101a, Tokyo, Japan). The pH levels were obtained using a digital pH meter (Crison Instruments, model GLP 21, Barcelona, Spain). Total acidity was measured through an acid-base titration, where the solution was titrated with a 0.1 N standard solution of sodium hydroxide and expressed as grams of citric acid per 100 mL.

### 2.4. Methanolic Extraction and UHPLC-Q-Orbitrap HRMS Analysis of Fruits

The extraction of polyphenols was carried out following a modified version of the method described by Iglesias-Carres et al. [14]. Briefly, 1 g of the lyophilized sample was placed into a 50 mL Falcon tube and extracted with 5 mL of an 80:20 (*v*/*v*) methanol–water solution containing 0.1% formic acid. The samples were vigorously mixed using a vortex mixer (ZX3; VEPL Scientific, Usmate, Italy) for 3 min, sonicated (LBS 1; Zetalab srl, Padua, Italy) for 10 min and stirred on a digital orbital shaker (SKO-D XL ARGOlab, Arezzo, Italy) for another 10 min. Afterward, the mixture was centrifuged at 5000 rpm for 5 min at 4 °C. The clear supernatant was collected, and the residue was re-extracted with another 5 mL of the same methanol–water mixture. This second supernatant was combined with the first, filtered through a 0.22 µm filter, and diluted tenfold for subsequent analysis using UHPLC-Q-Orbitrap HRMS [15]. The chemical characterization of polyphenols in the fruits was carried out as reported by Cirillo et al. [16].

### 2.5. Puree and Jam Preparation

Both apricot cultivars were processed to generate purees and jams. Briefly, apricots with the same ripeness washed with water and pitted were placed in a machine that cooks and homogenizes the food in a vacuum state (Roboqbo, Bentivoglio, Bologna, Italy). The vacuum was set at −850 mbar and the rotating cutter system at 3000 rpm, and thermal treatment was performed at 85 °C for 3 min to obtain the puree. For the jams, 1 kg of sucrose (Eridania, Bologna, Italy) was added to 2 kg of puree and the machine set with the vacuum at −980 mbar and the rotating cutter system at 500 rpm, and thermal treatment was performed at 60 °C until the jam was obtained having a final Brix° of 65%.

### 2.6. Color Analysis from Fruit to Jam

To evaluate the color change following the processing of the fresh product, color analysis of the fruits (skin and pulp), purees and jams was conducted using an IRIS Alpha-Mos electronic eye (Visual Analyzer, IRIS VA 400, Alpha M.O.S., Toulouse, France) and its AlphaSoft software (V14.2, Alpha MOS, Toulouse, France) as described by Falciano et al. [17]. To ensure consistent lighting and eliminate external influences, images of each sample were captured within a sealed light chamber (420 × 560 × 380 mm). This chamber was equipped with a dual top-and-bottom LED lighting system, preventing shadow formation. The LED system boasted a color temperature of 6700 K, a high color rendering index (CRI) of 98 (indicating excellent color reproduction fidelity), and a spectral power distribution closely resembling natural daylight (D65). A Basler ace GigE camera (acA2500-14gc, Ahrensburg, Germany) equipped with a 16 mm lens was employed for image acquisition. After calibration with a certified color scale, the samples were positioned on a removable white tray within the chamber, ensuring uniform light diffusion. Color measurements were conducted in triplicate using the internationally standardized CIELab color space. In this system, L* represents lightness (ranging from 0 for black to 100 for white), while a* and b* denote the green-red and blue-yellow color coordinates, respectively, each ranging from −100 to +100. Chroma Index (C*) and Redness Index were determined by using Equations (1) and (2), respectively [18]:

(1) $$C*=\sqrt{a^{2}+b^{2}}$$

(2) Redness Index=a/b

The images obtained were also processed as a color spectrum, representing the percentage of surface occupied by each color identified on a fixed scale of 4096 colors (Figures S1–S8).

### 2.7. Preparation of Methanolic Extracts for Spectrophotometer Analysis

The samples (1 g) were mixed with 25 mL of 70% (*v*/*v*) methanol, shaken at room temperature for 2 h and then centrifuged at 12,000× *g* for 15 min at 20 °C. The entire process was carried out twice. The supernatants obtained were combined and stored at −23 °C until the analysis.

### 2.8. Total Phenol Content

The total phenolic content (TPC) was determined according to Falciano et al. [19]. The extracts (50 μL) were mixed with 70 μL Folin–Ciocalteu reagent and 880 μL distilled water. The mixture was thoroughly mixed by vortex for 1 min and incubated for 5 min at room temperature. Subsequently, 530 μL distilled water and 70 μL of 7.5% (*w*/*v*) sodium carbonate were added to each tube and incubated at 45 °C in the dark for 15 min; then, the absorbance was measured at 760 nm using the UV-VIS spectrophotometer (V-730, JASCO International Co., Ltd., Hachioji, Japan—Sennincho). Gallic acid was used as standard, and the results were expressed as mg of Gallic Acid Equivalent (GAE)/g dry weight (DW).

### 2.9. Antioxidant Activity by ABTS, DPPH and FRAP Assays

The antioxidant activity was detected by using both the ABTS•+ and DPPH• assays according to the method of Falciano et al. [19]. Briefly, freshly prepared ABTS solution (7 mM) was incubated with 2.45 mM potassium persulfate (final concentration) in the dark at room temperature for 12–16 h to obtain the cationic radical (ABTS•+) before use. For the analyses, the ABTS•+ solution was diluted in 96% ethanol to an absorbance of 0.7 (±0.02) at 732 nm, then 1 mL of this solution was mixed with 25 μL of 70% methanol (blank) or sample extracts. The samples were incubated for 10 min at room temperature, and then the absorbance at 732 nm was measured.

The methanolic solution of DPPH• (0.1 mM) was freshly prepared, and then 950 μL was mixed with 50 μL of the sample extract or 50 μL of methanol (blank). The samples were incubated in the dark at room temperature for 1 h, and then the absorbance at 517 nm was measured.

The FRAP assay was conducted spectrophotometrically as described in previous studies [20]. A FRAP reagent was prepared by mixing a 10 mM TPTZ solution, a 20 mM ferric chloride solution, and a 0.3 M acetate buffer (pH 3.6) in a 1:1:10 ratio (*v*/*v*/*v*). The assay was performed using freshly prepared FRAP reagent. Shortly, 150 µL of the diluted sample was added to 2850 µL of the FRAP reagent. Absorbance was recorded after 4 min at 593 nm. Antioxidant activity by the ABTS, DPPH and FRAP assays was expressed as millimoles of Trolox equivalents/kg dry weight (DW).

### 2.10. Determination of Carotenoids

Total carotenoids were determined according to Fernandes et al. [21]. Briefly, about 1 g of sample was homogenized with 10 mL of distilled water for 2 min and mixed with 5 mL of hexane, vigorously stirred for 1 min, allowed to stand for 5 min for enhancement of mass transfer and then vigorously stirred for 1 min more. The supernatant was collected and analyzed spectrophotometrically at 452 nm and the concentration of total carotenoids expressed as micrograms of β-carotene equivalents/g of dry weight (μg/g).

### 2.11. Statistical Analysis

After confirming that all measurements followed a normal distribution using the Kolmogorov–Smirnov test and verifying the homogeneity of variances using Levene’s test, a one-way ANOVA was applied to each parameter to assess statistically significant differences among varieties. Tukey’s multiple range test (*p* = 0.05) was employed, as it is a widely used statistical method in agricultural research. Specifically, for the physico-chemical traits and the content of individual polyphenols in the fruits, a varietal comparison was conducted between the two analyzed cultivars. Additionally, for the evolution of colorimetric indices in the fruit pulp during processing into puree and jam, as well as for carotenoid content, total polyphenols, and antioxidant activity, one-way ANOVA analyses were performed. These analyses compared the two varieties and assessed changes within the same cultivar throughout the transformation process from fresh fruit to jam. The data were analyzed using Microsoft Excel and IBM^®^ SPSS Statistics, Package 6, version 23.0. The PCA (Principal Component Analysis) was conducted on the Chroma Index values, antioxidant activities, total polyphenols and carotenoids of the fruit, puree and jam from the two analyzed cultivars. The statistical package XLStat Version 2013 (New York, NY, USA) was used to perform all the analyses.

## 3. Results and Discussion

### 3.1. Physico-Chemical Characterization of the Apricot Fruits

**Figure 1:**
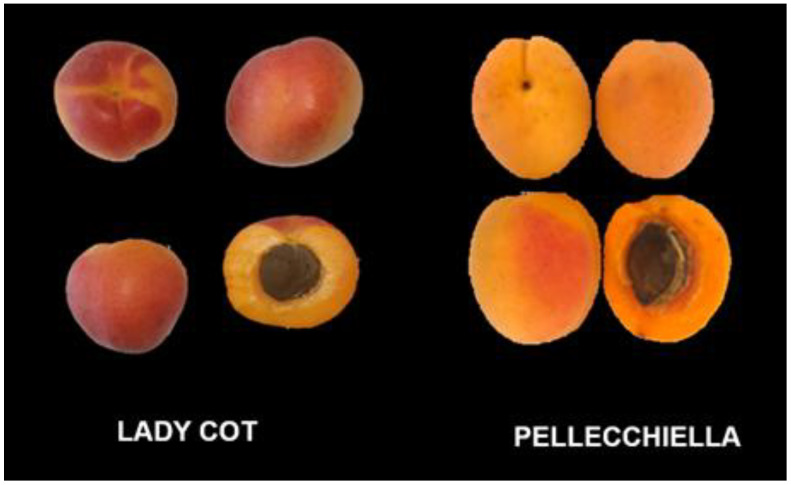
Photos of ‘Lady Cot’ and ‘Pellecchiella’ apricot cultivars.

This research focused on analyzing the morphological, qualitative and nutraceutical characteristics of the ‘Lady Cot’ and ‘Pellecchiella’ cultivars, evaluating also the stability of nutraceutical properties of the processed products, including puree and jam derived from these cultivars. Figure 1 shows the photos of the two apricot cultivars analyzed, confirming the phenotypic differences between them. Table 2 shows the physical traits of the fruits analyzed in the study.

Regarding fruit size, a significant difference was shown between the two, with higher values reported for the ‘Lady Cot’, whose fruit size was about 33.62% higher than that of the ‘Pellecchiella’. The ‘Lady Cot’ showed a greater transverse development than its other dimensions, classifying it as oblate due to its width exceeding its height. In contrast, the ‘Pellecchiella’ had more balanced proportions, with the polar and transverse dimensions being similar, making it fall into the ellipsoidal category according to the UPOV guidelines. Marked statistical differences emerged in pulp firmness, with the ‘Pellecchiella’ measuring 1.75 kg/0.5 cm^2^ compared to the ‘Lady Cot’ at 2.33 kg/0.5 cm^2^. This aligns with a previous study on Vesuvian apricot cultivars, where ‘Pellecchiella’ stood out for its softer flesh at around 0.84 kg/0.5 cm^2^ [8]. Such softness can be advantageous for transforming into products like purees and jams but may pose challenges for handling and transport in the fresh market. Rodrigues et al. [22] noted that consumers often prefer larger fruits for their visual appeal and perceived higher quality, suggesting that the physical attributes of ‘Lady Cot’ could make it more attractive in fresh markets. Firmness notably affects consumer acceptance by shaping perceptions of freshness and quality [23,24]. Consequently, maintaining optimal firmness during storage and distribution is crucial for minimizing post-harvest losses and preserving consumer satisfaction.

A key attribute is the color of the fruits’ epicarp, which reflects both their ripeness and carotenoid and anthocyanins content [25,26], playing a significant role in determining their market appeal. In industrial applications, the importance of color goes beyond aesthetics; it serves as a critical criterion for sorting and grading fruits, ensuring that only those meeting specific standards proceed to further stages of processing [27]. This dual importance highlights how color not only enhances the fruit’s appeal to consumers but also ensures consistency and efficiency in production, directly impacting both marketability and product quality.

Regarding the colorimetric parameters L*, a* and b* of the epicarp, significant differences were observed between the ‘Lady Cot’ and ‘Pellecchiella’ cultivars, providing valuable insights into their visual and chemical characteristics. The ‘Lady Cot’ cultivar exhibited a significantly lower L* value (52.88) compared to the ‘Pellecchiella’ (62.45), with high statistical significance (*p* < 0.05), appearing noticeably darker. For the a* parameter, representing the red-green chromatic component, the ‘Lady Cot’ showed a value of 39.37, slightly lower than the ‘Pellecchiella’ (41.13). Regarding the b* parameter, which represents the yellow-blue chromatic component, the ‘Lady Cot’ exhibited a markedly lower value (38.93) compared to the ‘Pellecchiella’ (61.91). These colorimetric differences highlight that ‘Lady Cot’ has a darker skin with less vibrant yellow-orange tones, while ‘Pellecchiella’ presents a lighter skin with richer and more vivid orange hues. This observation was later confirmed by the analysis of carotenoid content, which was significantly higher in the ‘Pellecchiella’.

The correlation between skin color and carotenoid content serves as an important indicator, not only for the nutraceutical properties but also for the aesthetic appeal and commercial value of the fruit. The Chroma Index (C*) represents the color intensity or saturation, with higher values indicating more vivid and vibrant coloration. In this study, the ‘Lady Cot’ displayed a lower Chroma value (55.38) compared to the ‘Pellecchiella’ (74.33), highlighting the latter’s significantly more saturated and intense coloration, as confirmed by the statistical significance (***). On the other hand, the Redness Index, which measures the proportion of red tones relative to other colors, was higher in the ‘Lady Cot’ (1.01) than in the ‘Pellecchiella’ (0.66). Although ‘Lady Cot’ fruits show weaker overall color saturation, they feature a stronger red component. Conversely, ‘Pellecchiella’ displays more vibrant color but a lower Redness Index, indicating a more yellow hue. These distinctions highlight the unique color profiles of both cultivars, which could affect consumer perception and choice.

**Figure 2:**
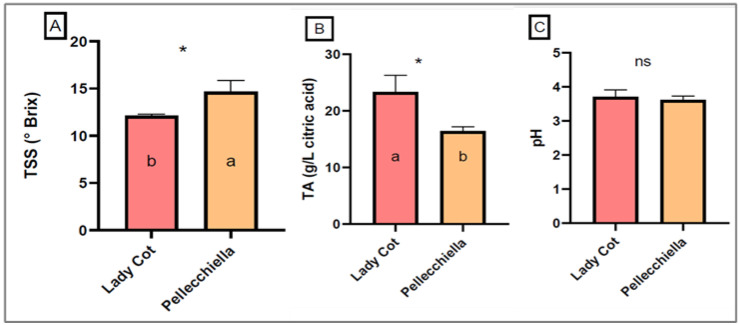
Qualitative traits (pH (**C**), TSS—total soluble solids (**A**) and TA—titrable acidity (**B**)) of the two analyzed apricot cultivars. Mean ± SD. Different letters indicate significant differences between the cultivars according to Tukey’s multiple range test (*p* < 0.05). Level of significance per the ANOVA is indicated as ns = not significant and * = *p* < 0.05.

However, the physical appearance of fruits alone is not sufficient to determine their overall quality. It is essential to investigate the chemical characteristics of fruits to obtain a more comprehensive understanding of consumer appeal. Indeed, such analyses provide crucial insights into factors like flavor, nutritional content and aroma, which significantly influence consumer preferences [28]. Figure 2 shows the chemical traits of the ‘Lady Cot’ and ‘Pellecchiella’, in terms of total soluble solids (TSS, °Brix), titratable acidity (TA, g/L of citric acid) and pH.

As for chemical traits, the ‘Lady Cot’ exhibited lower values of 12.00 °Brix for total soluble solids (TSS) compared to the ‘Pellecchiella’ (14.67 °Brix). However, the ‘Lady Cot’ showed a significantly higher titratable acidity (TA), with values 43.11% higher than those of the ‘Pellecchiella’. These results suggest that ‘Lady Cot’ has a more acidic profile with a less pronounced sweetness compared to ‘Pellecchiella’. While pH did not show significant differences between the cultivars, the higher acidity of ‘Lady Cot’ can influence its flavor, making it tangier. Vesuvian apricots, such as the ‘Pellecchiella’ cultivar, are known for their well-balanced sweetness and acidity, contributing to a richer and more appealing flavor. Ali et al. [29] noted that apricot cultivars grown in northern Pakistan exhibit TSS values ranging from 12.67 to 20.00 °Brix, while their acidity varies between 4.5 and 8.6, as shown in other comparative studies. For example, Melgarejo et al. [30] reported TSS values between 9.5 and 13 °Brix in apricots from southeastern Spain, making the ‘Pellecchiella’ cultivar particularly noteworthy due to its higher TSS content and superior flavor balance. In contrast, ‘Lady Cot’ has a slightly higher acidity and a less pronounced sweetness compared to the Vesuvian cultivar.

### 3.2. Determination of Polyphenol Content in the Apricot Fruits

Bioactive compounds, especially polyphenols, have attracted significant interest for their potent antioxidant and anti-inflammatory properties. These molecules, alongside carotenoids, help counter oxidative stress and reduce the risk of chronic diseases such as cardiovascular disorders and metabolic dysfunctions [31,32]. By neutralizing free radicals and safeguarding cellular integrity, they play a key role in overall health. Comparing the ‘Lady Cot’ and ‘Pellecchiella’ apricot cultivars highlights notable differences in polyphenol content, with the ‘Pellecchiella’ demonstrating a superior antioxidant and health-promoting profile (Table 3).

In this comparative analysis, the ‘Lady Cot’ exhibited significantly higher levels of quinic acid, with a 43% increase compared to the ‘Pellecchiella’. This suggests that ‘Lady Cot’ may possess unique health benefits associated with quinic acid, which is known for its potential role in metabolic and antioxidant functions. On the other hand, the ‘Pellecchiella’ demonstrated markedly higher levels of several key polyphenols. Catechin showed the most significant difference, with the ‘Pellecchiella’ containing 338% more than the ‘Lady Cot’. Similarly, epicatechin levels were 167% higher in the ‘Pellecchiella’, highlighting its superior profile in flavonoids associated with cardiovascular health [33]. Additionally, caffeic acid and ferulic acid were present in the ‘Pellecchiella’ at levels 55% and 33% higher, respectively, underscoring its enhanced antioxidant potential and its applicability in health and cosmetic products [34]. The differences in polyphenol composition between the two cultivars suggest that ‘Pellecchiella’, with its superior levels of bioactive compounds, offers a distinct advantage in functional food and nutraceutical applications aimed at promoting overall health and combating oxidative stress.

### 3.3. Evolution of Color, Carotenoids, Total Polyphenols, and Antioxidant Activity: From Fresh Fruit to Puree and Jam

A key objective of our study was to assess how the color, which is an important factor influencing consumer acceptability, and the nutraceutical components present in fresh fruit are altered during processing, transitioning from the fresh fruit to derived products such as puree and jam, and we also aimed to evaluate which of the two varieties had better presence of nutraceutical components throughout the transformation process. Table 4 provides an in-depth analysis of the evolution of L*, a* and b* values, C* and the Redness Index during the transformation process of the two apricot cultivars analyzed.

**Figure 3:**
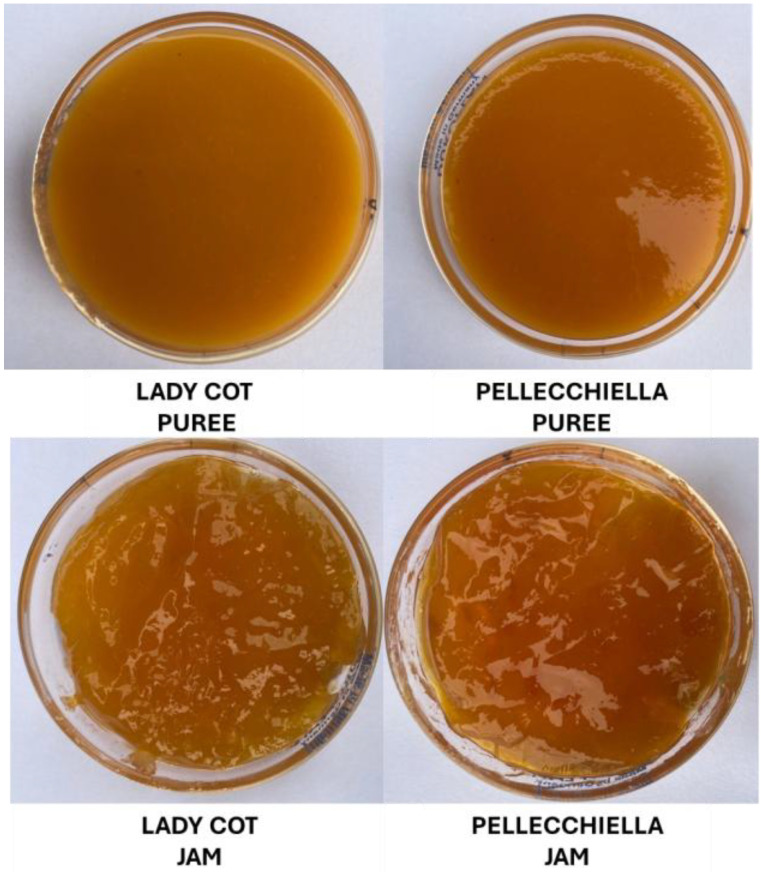
Photos of the purees and jams of the two cultivars analyzed.

The transformation process from fresh apricots to jam involves significant changes in colorimetric properties, reflecting alterations in pigmentation, oxidation and the effects of thermal processing. This analysis explores the evolution of lightness (L*), red-green component (a*), yellow-blue component (b*), the Chroma Index (C*) and the Redness Index at different processing stages. The L* parameter is crucial for assessing fruit quality, ripeness and commercial appeal, making it a key tool for producers and distributors in the agri-food sector. Our results show that, based on the values measured in the fruit pulp, this parameter increased during processing within the same cultivar, from fresh fruit to jam, by approximately 14.68% for the ‘Lady Cot’ and about 5.28% in the ‘Pellecchiella’. Significant differences between the two cultivars were observed only in the processed jam stage, with the ‘Pellecchiella’ apricot exhibiting a lower L* by about 8.72%. Regarding the a* parameter, the ‘Lady Cot’ cultivar showed no significant changes during processing. In contrast, the pulp of ‘Pellecchiella’ exhibited a notable decrease, with reductions of 37.2% in the puree and 32.88% in the jam. Additionally, the two cultivars displayed significant statistical differences across all three analyzed stages, with the ‘Pellecchiella’ showing higher values, particularly in relation to fruit pulp coloration. In both cultivars, the b* parameter increased noticeably during processing. For the ‘Lady Cot’, the b* rose by 28.88% from fresh fruit to jam. By contrast, the ‘Pellecchiella’ experienced an 8.98% increase at the puree stage, followed by a decrease at the jam stage, ultimately returning to values similar to the fresh pulp. C* indicates color saturation, with higher values signaling more intense and vibrant hues and lower values reflecting duller or grayer tones. The two cultivars followed contrasting trends during processing: the ‘Lady Cot’ showed a 26.17% increase in C* from fresh fruit to jam, while the ‘Pellecchiella’ remained stable from fruit to puree but decreased by 7.16% at the jam stage. One possible explanation for this trend is that, despite ‘Pellecchiella’ initially displaying a higher Redness Index due to its elevated polyphenol content, these pigments are more prone to degradation during processing [35]. Both cultivars experienced significant reductions in Redness Index values during processing. However, the ‘Pellecchiella’ maintained consistently higher values from the fresh fruit stage onward, a trend evident in both the puree and the jam. These contrasting trends reflect the interaction between each cultivar’s unique chemical composition and the physicochemical changes that occur during jam production [36] (Figure 3). Color is crucial to consumer appeal, influencing purchase decisions—particularly in fruits with a strong red hue [37]. While ‘Lady Cot’ may be preferred for its intense epicarp coloration when fresh, ‘Pellecchiella’s higher Redness Index in the pulp could make it more attractive in jam form.

During the fruit transformation process, not only does the color undergo significant changes but nutraceutical components such as polyphenols, carotenoids and antioxidant activity are also affected. Table 5 illustrates the evolution of all these parameters, tracking the transition from fresh fruit to puree and finally to jam.

As previously reported, the orange color of apricots is a distinctive feature primarily attributed to the presence of carotenoids, a class of natural pigments that not only impart yellow, orange and red hues to fruits but also provide significant nutritional benefits, including a high antioxidant capacity [38]. Among the most representative carotenoids in apricots is β-carotene, a well-known precursor of vitamin A, which plays a key role in orange pigmentation. Ruiz et al. [39] have shown that β-carotene content can vary significantly among varieties, directly affecting the intensity of flesh and skin color.

The ‘Lady Cot’ exhibited lower carotenoid levels compared to the ‘Pellecchiella’ across all processing stages, with a statistically significant difference between the two cultivars (*p* < 0.05). In the fresh fruit, the carotenoid content of the ‘Lady Cot’ was 29.4% lower than that of the ‘Pellecchiella’. During the transformation into puree, a slight decrease in carotenoid levels was observed for both cultivars, with the ‘Lady Cot’ decreasing to 1380.20 μg/g dw compared to 1930.66 μg/g dw for the ‘Pellecchiella’. Finally, in the jam, the carotenoid content decreased further in both cultivars, reaching 1280.55 μg/g dw for the ‘Lady Cot’ and 1809.77 μg/g dw for the ‘Pellecchiella’. The carotenoid content remained relatively stable from fresh fruit to puree in both cultivars, suggesting good retention during the initial processing stage. However, when transitioning from puree to jam, a pronounced decrease was observed, likely due to the thermal processing required for jam preparation. These results highlight that, despite the loss of carotenoids during processing, the ‘Pellecchiella’ cultivar consistently maintained significantly higher levels compared to the ‘Lady Cot’ at every stage. This difference suggests that ‘Pellecchiella’ may offer greater nutritional and functional benefits associated with carotenoids, such as antioxidant potential and support for visual and skin health [40], making it particularly interesting for applications in the food and nutraceutical sectors.

The higher carotenoid content in ‘Pellecchiella’ is also reflected in its vibrant orange coloration, as previously observed. Carotenoids play a significant dual role, benefiting both the plant and human health [41]. In plants, carotenoids not only contribute to their striking color but also serve as crucial protectants [42]. They act as potent antioxidants, safeguarding cells from oxidative damage caused by environmental stressors, including UV radiation [43]. For human health, carotenoids are equally indispensable. The carotenoid content in the ‘Pellecchiella’ (2010.45 μg/g dw) was significantly higher compared to international cultivars, including those cultivated in the Baranja region of Croatia, as analyzed by Dragović-Uzelac et al. [38]. In particular, their study reported carotenoid values of approximately 683 μg/g dw for ‘Keckemetska Ruza’, 1478 μg/g dw for ‘Madjarska Najbolja’ and 1064 μg/g dw for ‘Velika Rana’, varying according to the stage of maturity and geographical region. This comparison highlights the exceptional nutraceutical potential of the ‘Pellecchiella’ apricot. This superior trend of ‘Pellecchiella’ is also confirmed by a comparison with the Spanish cultivars analyzed by Ruiz et al. [44], which reported significantly lower carotenoid levels. Another study by Zhou et al. [45] revealed that carotenoid metabolism, influenced by specific genes, plays a crucial role during fruit development, shaping the diversity of apricot color profiles. Additionally, apricots are a rich source of phytoene and phytofluene, precursor carotenoids having unique properties, as reported in a study by Gecer et al. [46], which analyzed various apricot accessions and highlighted a wide variability in carotenoid content. These pigments are not only essential for color but also contribute to the perception of quality and the fruits’ market appeal. Differences in total carotenoid content between white- and orange-skinned cultivars, for instance, have also been described by Dragovic-Uzelac et al. [38], emphasizing the importance of environmental and genetic factors in determining the color and nutritional profile of apricots. Additionally, the antioxidant activity of the ‘Pellecchiella’ was comparable to or higher than that of other Vesuvius cultivars, such as ‘Vollese’ (34.04 ± 1.50 mg GAE/100 g and 1.76 ± 0.02 µM TE/g) and ‘Preveta Bella’ (31.32 ± 2.47 mg GAE/100 g). However, when compared to other fruits known for their robust antioxidant properties, such as berries and pomegranates, the antioxidant capacity of apricots is relatively lower due to the absence of anthocyanins, which are abundant in those fruits [47]. These findings highlight the exceptional nutraceutical value of the ‘Pellecchiella’ cultivar, emphasizing its potential health benefits and its competitive position among international apricot cultivars.

Also, polyphenol content and antioxidant activity, key health-beneficial parameters, were compared between the two apricot cultivars and evaluated throughout processing (Table 5). In the ‘Lady Cot’, the polyphenol levels dropped from 1715.90 μg/g dw in the fresh fruit to 1300.30 μg/g dw in the puree (−24.2%) and to 669.66 μg/g dw in the jam (−61.0% total). The ‘Pellecchiella’ showed higher levels throughout, decreasing from 2047.65 μg/g dw in the fresh fruit to 1766.33 μg/g dw in the puree (−13.7%) and 800.99 μg/g dw in the jam (−60.9% total). Overall, processing led to an over 60% reduction in polyphenols, with the ‘Pellecchiella’ demonstrating superior antioxidant activity, consistent with earlier findings on its exceptional properties [8]. Antioxidant activities (ABTS, DPPH and FRAP) decreased significantly in both the ‘Lady Cot’ and the ‘Pellecchiella’ during processing. For ABTS, the ‘Lady Cot’ dropped by 55.9% and the ‘Pellecchiella’ by 55.7% from fresh fruit to jam. For DPPH, the ‘Lady Cot’ declined by 44.9% and the ‘Pellecchiella’ by 59.7%. FRAP showed reductions of 55.6% for the ‘Lady Cot’ and 55.9% for the ‘Pellecchiella’. At all stages, the ‘Pellecchiella’ maintained higher antioxidant activity. The largest losses occurred from puree to jam, likely due to heat and oxidation, with total reductions exceeding 50% in both cultivars by the jam stage. Both in polyphenol content and antioxidant activity, the largest losses occurred from puree to jam, likely due to heat and oxygen exposure. It is known that in the production of jams, high heat treatment conditions directly influence the sensory properties and nutritional constituents of jams, and the processes applied can significantly reduce their nutritional value [48]. Bioactive compounds such as carotenoids and polyphenols, and their overall antioxidant potential, are heat-sensitive components whose rate of deterioration varies with the applied process conditions [49]. Polyphenols and carotenoids exhibit markedly different behaviors during food processing due to factors such as temperature, pH, vacuum cooking and sugar addition, which affect their stability and absorption [50,51,52]. In general, carotenoids become more bioavailable after thermal treatments but degrade more rapidly in acidic environments, whereas polyphenols are susceptible to breakdown under high temperatures or alkaline conditions. These patterns align with the observed losses of polyphenols (approximately 61% in both cultivars) and carotenoids (with higher values for the ‘Pellecchiella’) during the transition from fresh fruit to puree and jam, findings consistent with studies reporting total polyphenol reductions in fruit-based jams ranging from roughly 42% to 93%. For instance, Rababah et al. [53], who investigated phenolic compound evolution in strawberry, black cherry, apricot, fig and orange jams, documented polyphenol losses as high as 72% in apricot jam. This figure exceeds the 61% decrease reported here, likely because the vacuum cooking method employed in the present study and its relatively short processing times can help mitigate both enzymatic and non-enzymatic degradation. Similarly, recent research on tropical fruits confirms significant carotenoid depletion under thermal treatment, principally caused by heat exposure and oxidation [54]. Furthermore, Drogoudi et al. [55] observed a 48.3% reduction in total polyphenol content in peach jam versus fresh fruit, with antioxidant capacity dropping even more sharply than total polyphenol levels. Collectively, these findings underscore the importance of optimizing processing parameters and favoring less aggressive methods such as vacuum cooking to preserve the nutritional efficacy of processed products and maximize the retention of bioactive compounds.

Regarding the carotenoid content, the trend was confirmed by Abd-Elnoor [56] where no significant differences were detected between fresh apricot fruit and puree, while there was a 14% reduction at the jam stage. Giuffrida et al. [57] also observed that carotene levels in peaches remained stable throughout processing into juices, jams and other products, regardless of the treatment methods. Hwang et al. [58] demonstrated that elevated temperatures and extended baking durations facilitate the release of carotenoids from tomatoes. Consistent with this, numerous studies have documented a significant increase in β-carotene content within pumpkin following cooking. This phenomenon can be attributed to the fact that heat enhances the bioavailability of these compounds [59].

### 3.4. Principal Component Analysis of Bioactive Parameters and Color from Whole Fruit to Puree and Jam

**Figure 4:**
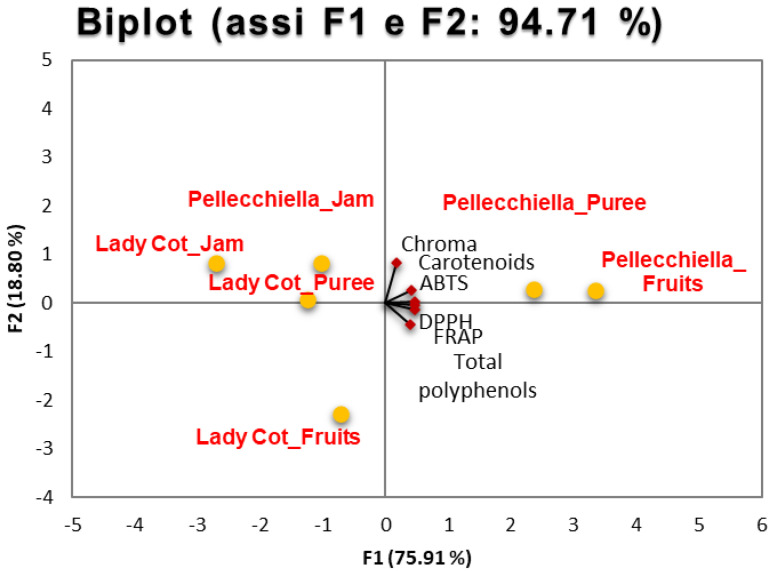
PCA (Principal Component Analysis) of the nutraceutical and colorimetric parameters analyzed in the fruits, purees and jams of the two cultivars studied.

The PCA biplot reveals distinct differences between the ‘Pellecchiella’ and ‘Lady Cot’ apricot cultivars (Figure 4), with the first two components explaining 94.71% of the total variance (F1: 75.91%, F2: 18.80%). This high variance capture indicates that the selected variables effectively represent the nutritional and qualitative diversity of the cultivars. The analysis was performed on total polyphenols, antioxidant activities (ABTS, DPPH and FRAP), carotenoids and Chroma Index parameters to investigate variation patterns and correlations across fresh fruits, purees and jams.

The ‘Pellecchiella’ demonstrates greater variability, with jams closer to F2, indicating chemical transformations from thermal processing (e.g., carotenoid degradation and Maillard reaction products). This cultivar shows superior antioxidant properties and pigmentation, linked to high carotenoid content, suggesting potential for functional foods, nutraceuticals and natural colorants. In contrast, the ‘Lady Cot’ products are more tightly clustered, showing consistent chemical profiles across processed forms. This indicates a lower impact of processing on their nutraceutical properties, attributed to stable pigment composition and lower initial antioxidant activity. ‘Lady Cot’ is positioned as a cultivar with sensory appeal, suitable for fresh consumption and confectionery products. ‘Pellecchiella’ is highlighted for its nutritional superiority due to high carotenoid content and antioxidant activity, making it suitable for health-focused applications. ‘Lady Cot’ maintains sensory stability across processing, favoring its market potential for products requiring uniform flavor profiles.

The PCA analysis demonstrates the distinct nutritional profiles and processing impacts on the two cultivars. It supports targeted marketing strategies by positioning ‘Pellecchiella’ as a premium health-oriented product, while ‘Lady Cot’ is marketed for its sensory appeal and versatility in both fresh and processed forms. This research provides valuable insights for product development and consumer segmentation based on the nutritional and functional potential of these apricot cultivars.

## 4. Conclusions

This study comparing the international ‘Lady Cot’ and the native Vesuvian ‘Pellecchiella’ apricot cultivars revealed significant differences in their physico-chemical and nutraceutical characteristics, both in fresh fruits and processed products such as purees and jams. While ‘Lady Cot’ stands out for its larger size, greater Redness Index of epicarp, and pulp firmness that enhance transportability and consumer appeal, ‘Pellecchiella’ excels in terms of sweetness, with higher total soluble solids (TSS) and lower acidity, making it particularly appealing in terms of flavor. Furthermore, the high carotenoid content observed in ‘Pellecchiella’ is confirmed by the pronounced orange hue of its skin, highlighting the strong relationship between the visual appearance and nutritional composition of the fruit. From a nutraceutical perspective, the ‘Pellecchiella’ showed superior levels of polyphenols, antioxidant activity and carotenoids compared to the ‘Lady Cot’, both in fresh fruit and throughout processing. Although processing led to a reduction in total polyphenols and antioxidant properties for both cultivars, the ‘Pellecchiella’ retained its nutraceutical properties more effectively, further emphasizing its value as a functional food. These findings highlight the importance of starting with high-quality raw materials to achieve a superior final product, as demonstrated by ‘Pellecchiella’, where the exceptional quality of the fruit translates into processed products of outstanding value. Based on our results, consumers are likely to prefer ‘Lady Cot’ fruits due to their larger size and redder epicarp, while ‘Pellecchiella’s stronger red component in processed products may enhance its appeal. From a commercial perspective, the superior nutraceutical and sensory traits of ‘Pellecchiella’ suggest opportunities for developing premium products that cater to health-oriented and gourmet markets. Moreover, future research could focus on assessing compound stability under different storage conditions or expanding the range of derived products—such as functional beverages or snack formulations—to fully leverage ‘Pellecchiella’s outstanding qualities. These findings highlight the need to promote the ‘Pellecchiella’ cultivar for its superior nutrition and health benefits while exploring other Vesuvian cultivars to boost the regional apricot industry and meet the demand for high-quality functional foods.

## Figures and Tables

**Table 1:** Origin and description of the two apricot cultivars analyzed.

Characteristic	Lady Cot	Pellecchiella
Origin	Selected in France	Native to the Vesuvian area
Fruit shape	Round, symmetrical	Oval, slightly elongated
Skin color	Bright orange with red streaks	Intense yellow
Flesh color	Light orange, firm	Yellow, juicy
Texture	Firm and crunchy	Medium, slightly fibrous
Taste	Sweet-tart, well-balanced	Sweet, aromatic
Fruit size	Medium	Small to medium
Main use	Fresh consumption, processing	Fresh consumption, jam-making

**Table 2:** Physical traits of the fruits of the two analyzed apricot cultivars: fruit weight (g), firmness (kg/0.5 cm^2^), polar diameter (mm), equatorial diameter (mm), transversal diameter (mm), epicarp color coordinates L*, a*, b*, Chroma Index (C*) and Redness Index.

Physical Trait	Lady Cot	Pellecchiella	Significance
Fruit Weight (g)	61.04 ± 8.33 b	45.68 ± 8.85 a	***
Firmness (kg/0.5 cm^2^)	2.33 ± 0.18 a	1.75 ± 0.22 b	***
Polar Diameter (mm)	48.92 ± 2.77 a	47.03 ± 2.95 b	***
Equatorial Diameter (mm)	46.03 ± 2.76 b	40.77 ± 3.72 a	***
Transversal Diameter (mm)	50.50 ± 3.08 a	47.57 ± 3.29 b	**
L*	52.88 ± 2.59 b	62.45 ± 3.59 a	***
a*	39.37 ± 7.59 b	41.13 ± 1.59 a	***
b*	38.93 ± 8.59 b	61.91 ± 1.59 a	***
C*	55.38 ± 1.74 b	74.33 ± 2.11 a	***
Redness Index	1.01 ± 0.04 a	0.66 ± 0.07 b	***

Mean ± SD. The same letter indicates no significant differences between the cultivars according to Tukey’s multiple range test (*p* < 0.05). Level of significance per the ANOVA is indicated as ** = *p* < 0.01 and *** = *p* < 0.001.

**Table 3:** Polyphenol profile of the two analyzed cultivars; all values are expressed as μg/g.

Polyphneol (μg/g)	Lady Cot	Pellecchiella	Significance
quinic_acid	346.42 ± 49.06 a	241.78 ± 32.41 b	**
protocatechiuc_acid	1.51 ± 0.22	1.27 ± 0.29	ns
caffeic_acid	16.60 ± 3.93 b	25.67 ± 4.26 a	*
epicatechin	30.34 ± 7.22 b	80.94 ± 14 a	***
chlorogenic_acid	641.14 ± 35.37	770.65 ± 147.96	ns
catechin	76.84 ± 5.85 b	336.33 ± 62.84 a	***
p-coumaric	10.10 ± 4.61	13.37 ± 0.90	ns
siringic_acid	24.38 ± 2.94	25.9 ± 0.72	ns
ferulic_acid	58.64 ± 9.62 b	77.87 ± 11.02 a	*
rutin_hydrate	508.06 ± 135.32	471.69 ± 49.55	ns
isorhamnetin-3-rutinoside	1.89 ± 0.11	2.18 ± 0.44	ns
myricitrin	4.64 ± 1.84	3.44 ± 0.39	ns

Mean ± SD. Different letters indicate significant differences between the cultivars according to Tukey’s multiple range test (*p* < 0.05). Level of significance per the ANOVA is indicated as: ns = not significant; * = *p* < 0.05; ** = *p* < 0.01; *** = *p* < 0.001.

**Table 4:** Evolution of the L*, a* and b* values, C* and the Redness Index during the transformation process from fruit to jam in the two analyzed apricot cultivars.

	Color Value	Lady Cot	Pellecchiella
Fruits: Mesocarp	L*	63.66 ± 3.16 b	63.77 ± 3.85 b
	a*	23.31 ± 1.13 a	44.79 ± 2.21 a *
	b*	58.79 ± 2.90 b	70.02 ± 3.50 b *
	C*	63.24 ± 3.13 b	83.13 ± 2.19 a *
	Redness Index	0.40 ± 0.03 b	0.64 ± 0.06 a *
Puree Apricot	L*	71.39 ± 1.28 a	73.07 ± 1.55 a
	a*	24.88 ± 1.03 a	28.07 ± 1.43 b *
	b*	74.38 ± 1.76 a	76.31 ± 2.00 a
	C*	78.42 ± 1.40 a	81.30 ± 1.85 a
	Redness Index	0.33 ± 0.03 a	0.37 ± 0.05 b
Jam Apricot	L*	73.00 ±1.54 a	67.14 ± 1.61 b *
	a*	25.04 ±1.95 a	30.06 ± 1.75 b *
	b*	75.77 ± 1.98 a	71.34 ± 1.27 b *
	C*	79.79 ± 1.61 a	77.17 ± 0.93 b
	Redness Index	0.33 ± 0.02 a	0.42 ± 0.06 b *

Mean ± SD. Different letters indicate significant differences within the same cultivar for the same parameter during the transformation process according to Tukey’s multiple range test (*p* < 0.05). An asterisk (*) denotes a statistically significant difference (*p* < 0.05) between the two cultivars for the same parameter.

**Table 5:** Evolution of Carotenoids (μg/g dw), total polyphenols (μg/g dw), and antioxidant activities by ABTS, DPPH and FRAP (mmol Trolox/kg dw) in the two apricot cultivars during processing from fresh fruit to puree and jam.

		Lady Cot	Pellecchiella
Fruit	Carotenoids (μg/g dw)	1420.21 ± 60.07 a	2010.45 ± 116.27 a *
	Total polyphenols (μg/g dw)	1715.90 ± 39.43 a	2047.65 ± 36.79 a *
	ABTS (mmol trolox/kg)	19.15 ± 3.08 a	42.08 ± 1.61 a *
	DPPH (mmol trolox/kg)	9.47 ± 1.45 a	15.53 ± 1.72 a *
	FRAP (mmol trolox/kg)	15.73 ± 0.99 a	30.99 ± 3.17 a *
Puree	Carotenoids (μg/g dw)	1380.20 ± 20.55 a	1930.66 ± 30.44 a *
	Total polyphenols (μg/g dw)	1300.30 ± 55.87 b	1766.33 ± 44.78 b *
	ABTS (mmol trolox/kg)	14.52 ± 3.22 b	40.22 ± 7.31 b *
	DPPH (mmol trolox/kg)	7.24 ± 1.23 b	13.44 ± 4.32 b *
	FRAP (mmol trolox/kg)	14.04 ± 2.45 b	25.99 ± 3.22 b *
Jam	Carotenoids (μg/g dw)	1280.55 ± 17.95 b	1809.77 ± 33.57 b *
	Total polyphenols (μg/g dw)	669.66 ± 22.37 c	800.99 ± 20.99 c *
	ABTS (mmol trolox/kg)	8.44 ± 1.97 c	18.66 ± 2.67 c *
	DPPH (mmol trolox/kg)	5.22 ± 0.87 c	6.25 ± 2.35 c *
	FRAP (mmol trolox/kg)	6.99 ± 1.54 c	13.67 ± 2.55 c *

Mean ± SD. Different letters indicate significant differences within the same cultivar for the same parameter during the transformation process according to Tukey’s multiple range test (*p* < 0.05). An asterisk (*) denotes a statistically significant difference (*p* < 0.05) between the two cultivars for the same parameter.

## Data Availability

The original contributions presented in this study are included in the article/Supplementary Materials. Further inquiries can be directed to the corresponding authors.

## Supplementary Materials

The following supporting information can be downloaded at https://www.mdpi.com/article/10.3390/foods14060945/s1. Figure S1: Color spectra of the ‘Lady Cot’ epicarp showing the proportion (percentage of surface) of each unique color measured in a 4096-color space if greater than 1%; Figure S2: Color spectra of the Pellecchiella epicarp showing the proportion (percentage of surface) of each unique color measured in a 4096-color space if greater than 1%; Figure S3: Color spectra of the ‘Lady Cot’ esocarp showing the proportion (percentage of surface) of each unique color measured in a 4096-color space if greater than 1%; Figure S4: Color spectra of the ‘Pellecchiella’ esocarp showing the proportion (percentage of surface) of each unique color measured in a 4096-color space if greater than 1%; Figure S5: Color spectra of the ‘Lady Cot’ puree showing the proportion (percentage of surface) of each unique color measured in a 4096-color space if greater than 1%; Figure S6: Color spectra of the ‘Pellecchiella’ puree showing the proportion (percentage of surface) of each unique color measured in a 4096-color space if greater than 1%; Figure S7: Color spectra of the ‘Lady Cot’ jam showing the proportion (percentage of surface) of each unique color measured in a 4096-color space if greater than 1%; Figure S8: Color spectra of the ‘Pellecchiella’ jam showing the proportion (percentage of surface) of each unique color measured in a 4096-color space if greater than 1%.
