# Supplementary material for: Comparative Evaluation of Qualitative and Nutraceutical Parameters in Fresh Fruit and Processed Products of ‘Lady Cot’ and Vesuvian ‘Pellecchiella’ Apricot Cultivars
Source: Foods. 2025 Mar 10;14(6):945. doi: 10.3390/foods14060945 (PMC11941052; doi:10.3390/foods14060945)

Figure S1.

Color spectra of the Lady Cot epicarp showing the proportion (percentage of surface) of each unique color measured in a 4096-color space if greater than 1%.

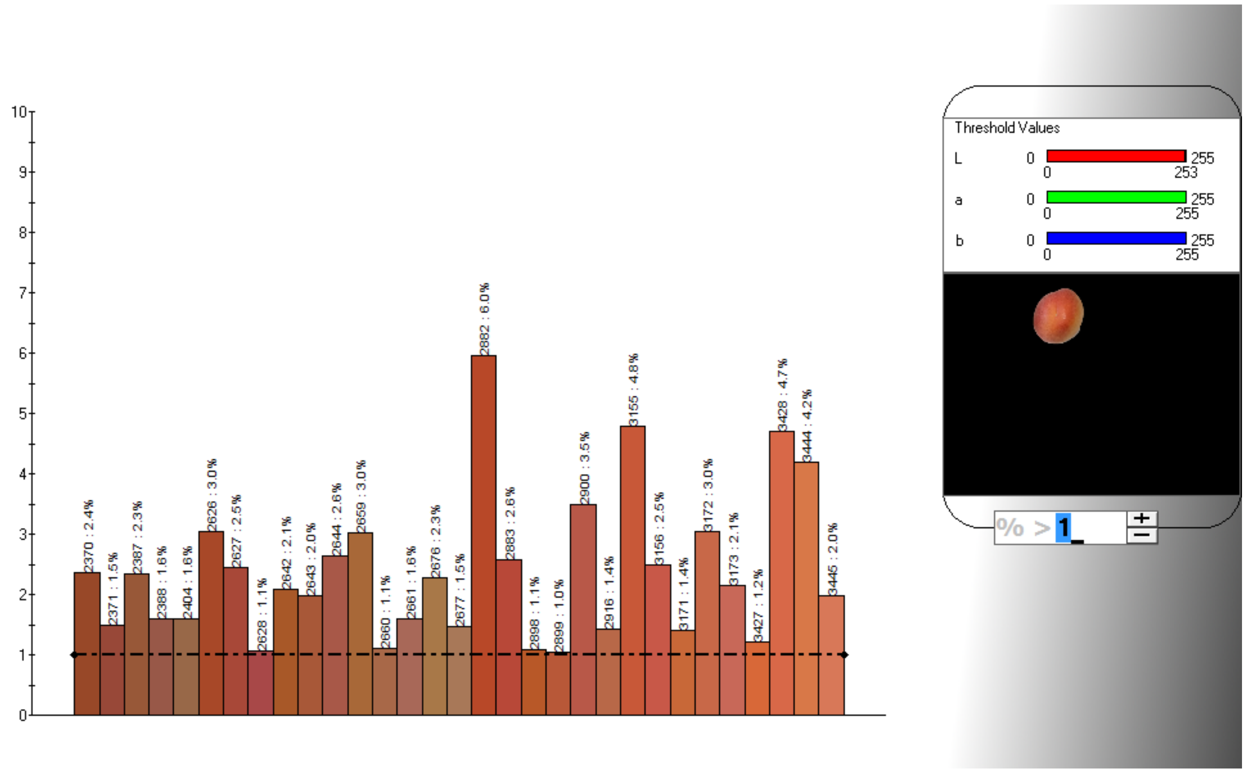

Figure S2

Color spectra of the Pellecchiella epicarp showing the proportion (percentage of surface) of each unique color measured in a 4096-color space if greater than 1%.

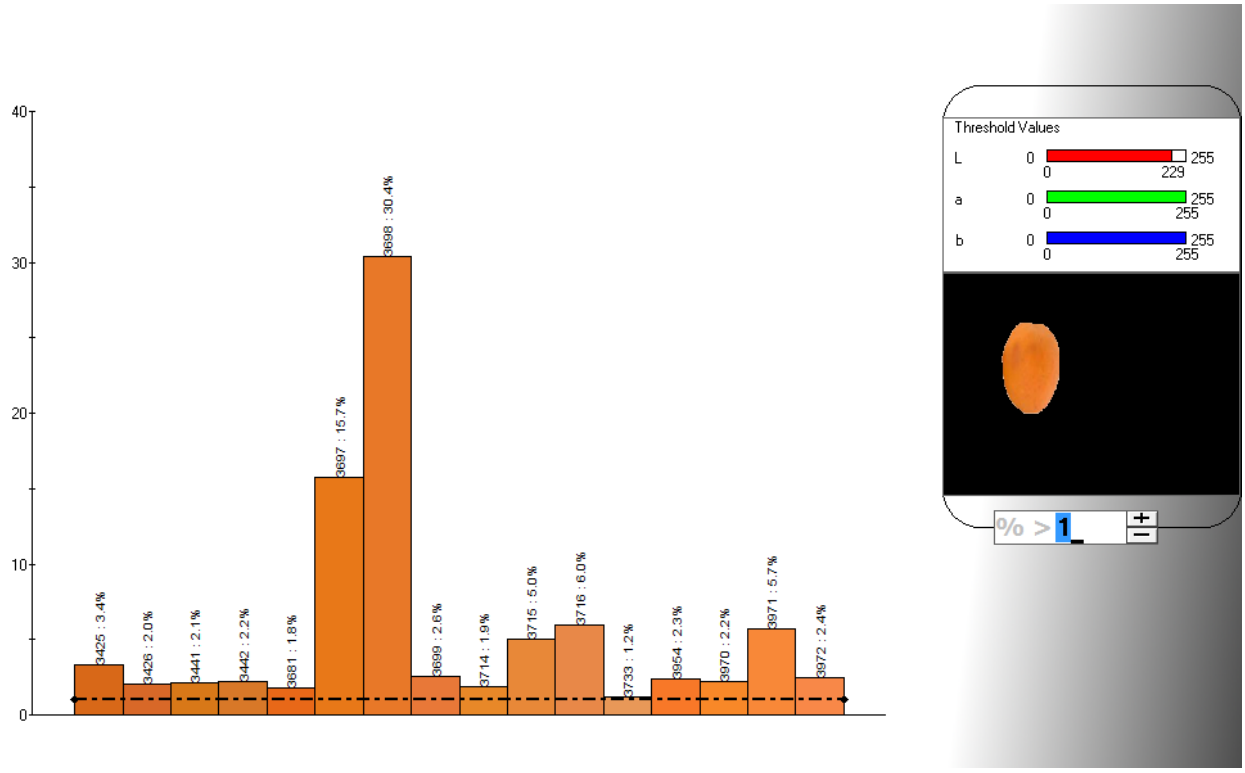

Figure S3

Color spectra of the Lady Cot esocarp showing the proportion (percentage of surface) of each unique color measured in a 4096-color space if greater than 1%.

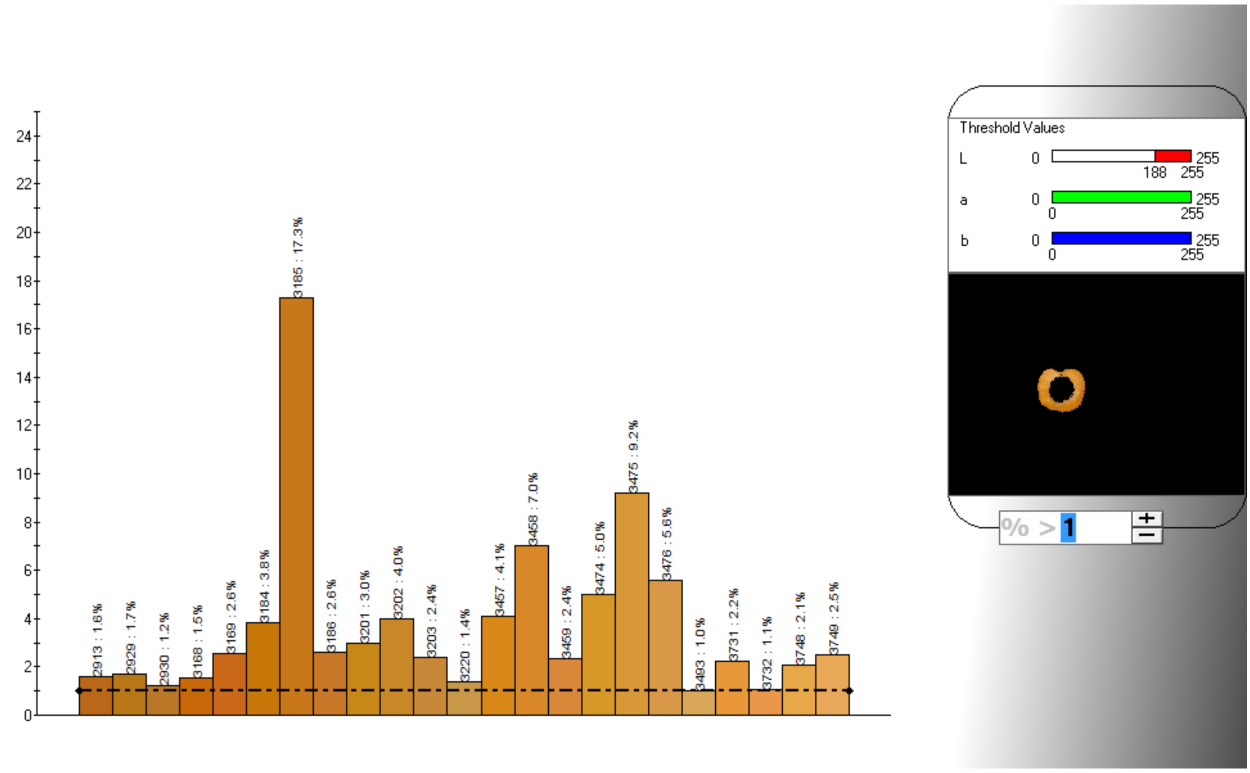

Figure S4

Color spectra of the Pellecchiella esocarp showing the proportion (percentage of surface) of each unique color measured in a 4096-color space if greater than 1%.

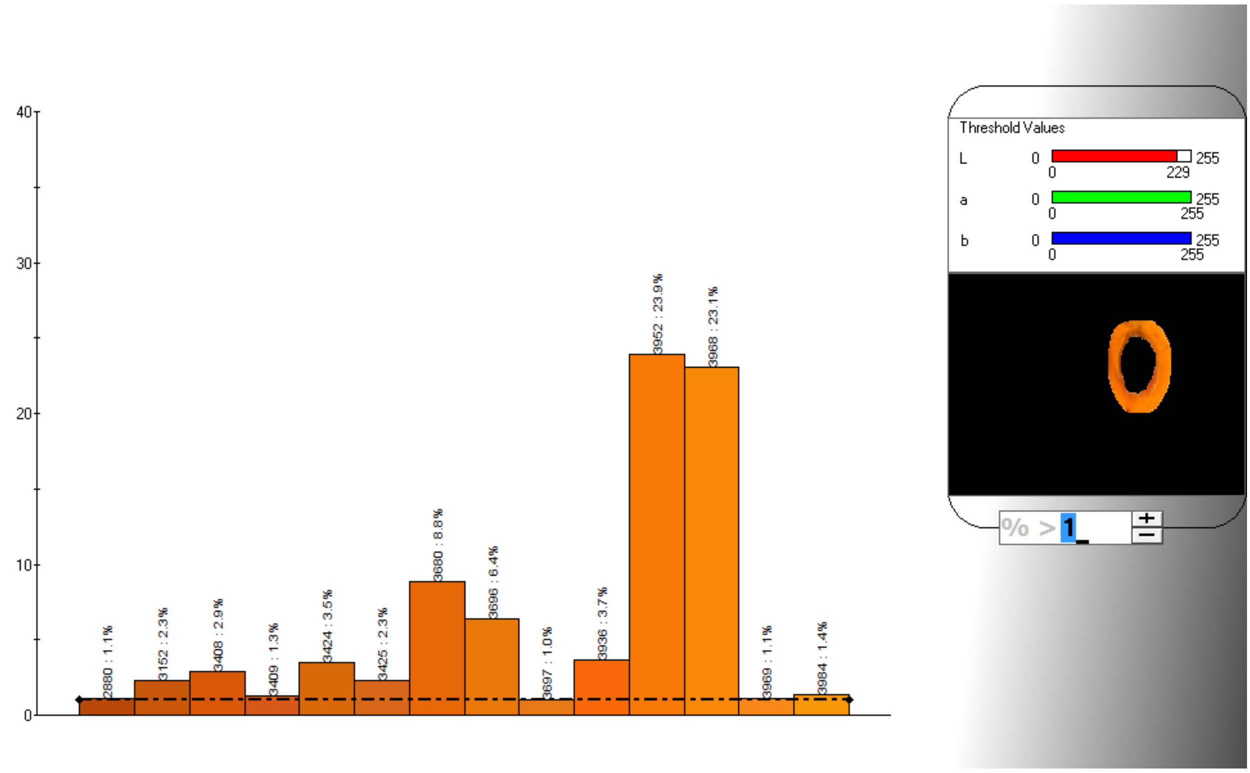

Figure S5

Color spectra of the Lady Cot puree showing the proportion (percentage of surface) of each unique color measured in a 4096-color space if greater than 1%.

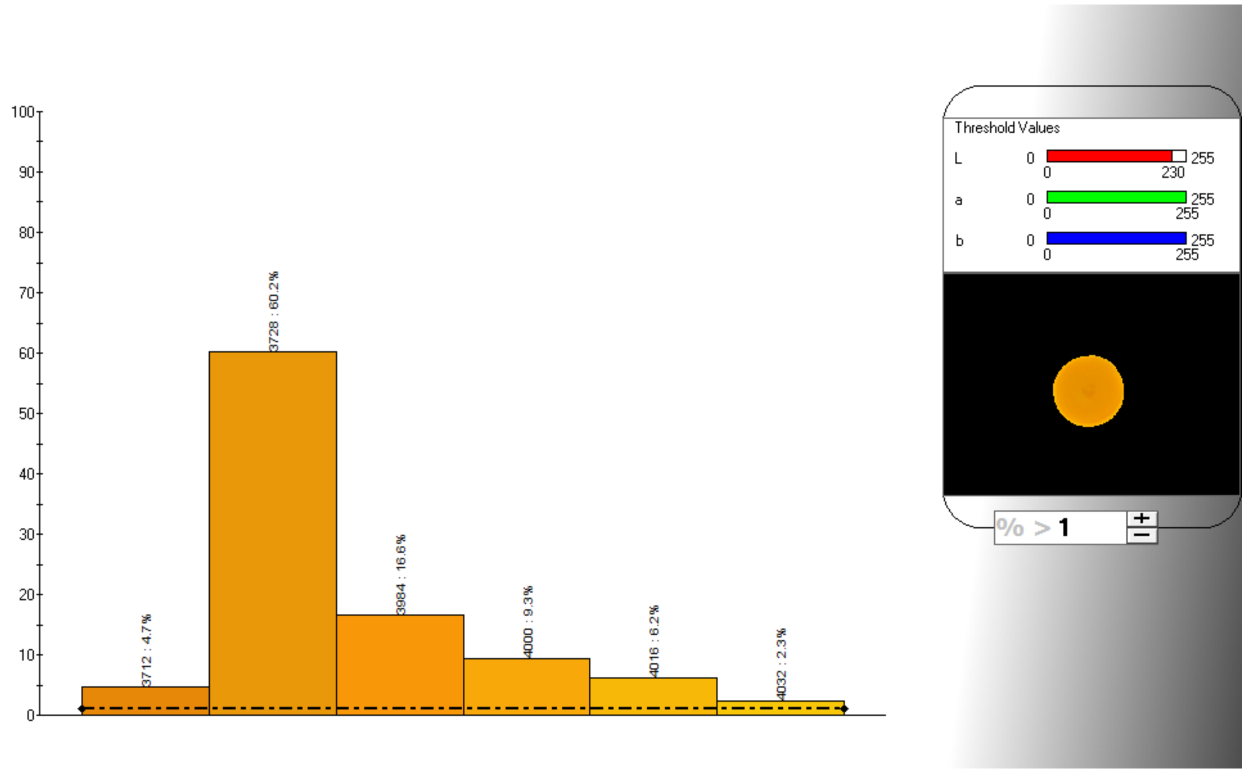

Figure S6

Color spectra of the Pellecchiella puree showing the proportion (percentage of surface) of each unique color measured in a 4096-color space if greater than 1%.

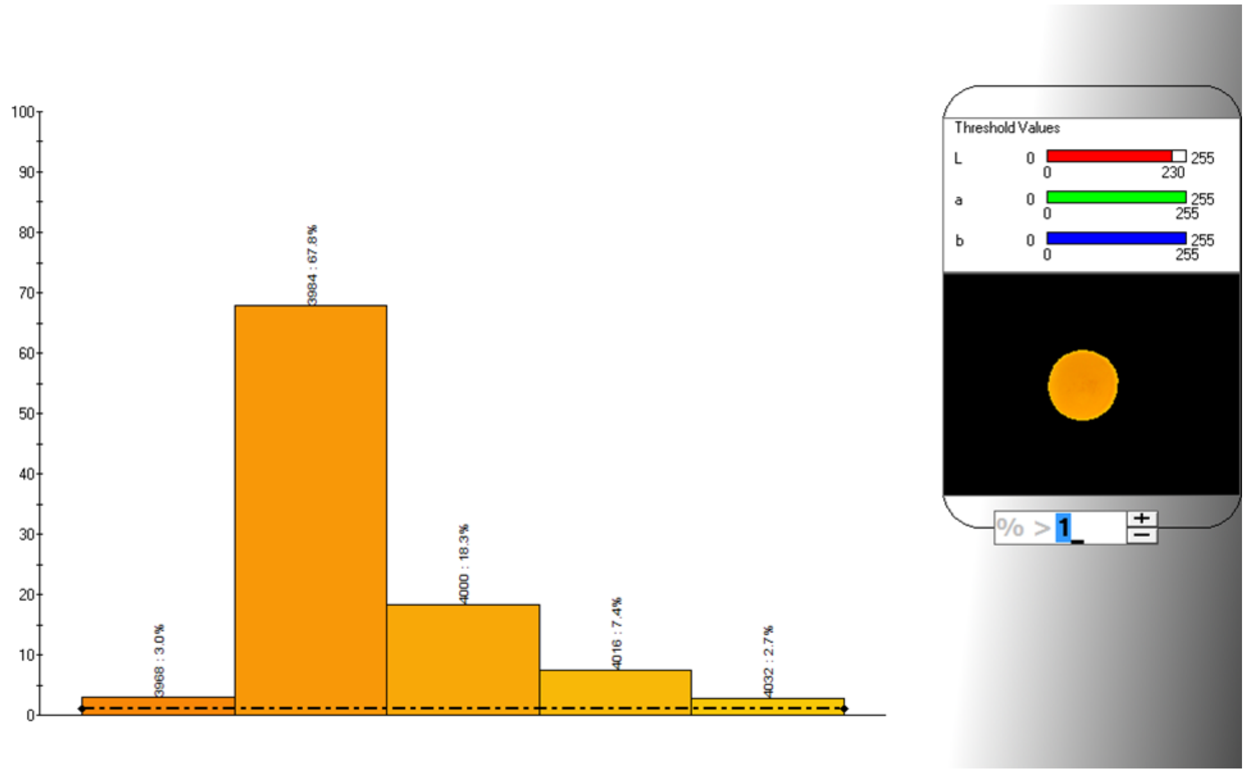

Figure S7

Color spectra of the Lady Cot jam showing the proportion (percentage of surface) of each unique color measured in a 4096-color space if greater than 1%.

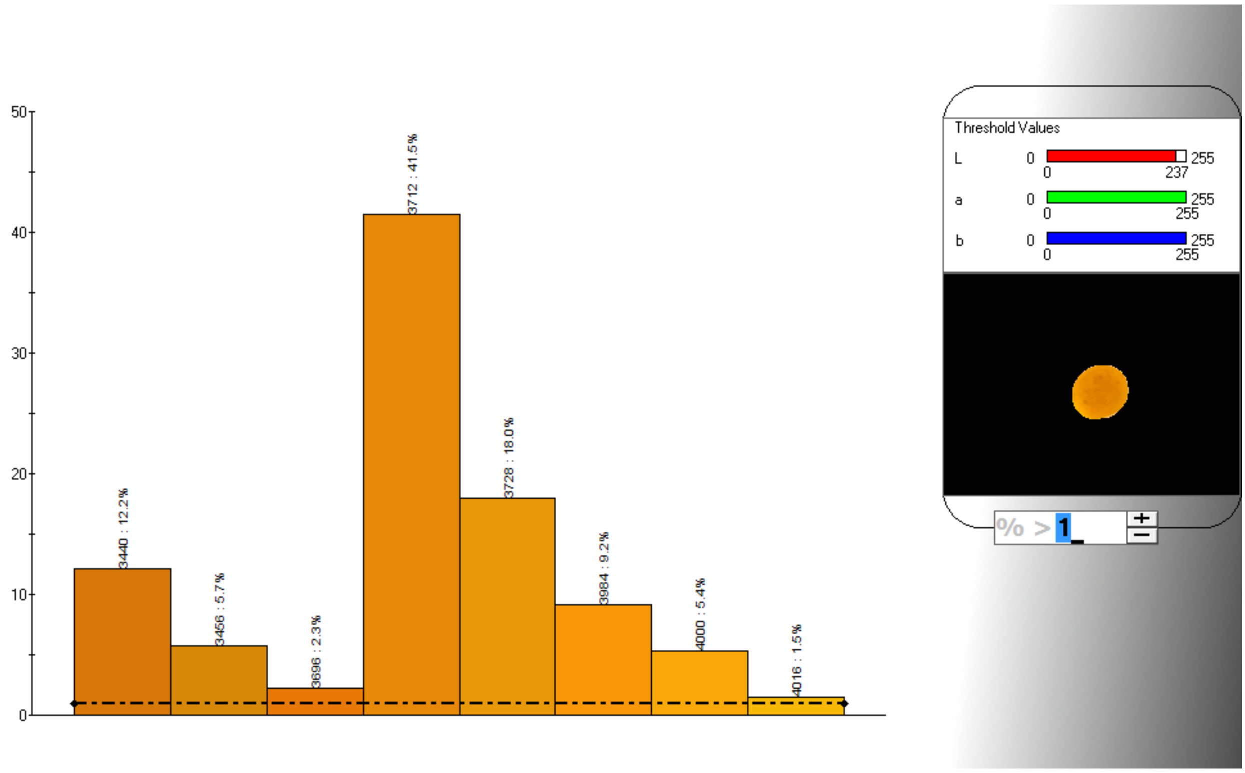

Figure S8

Color spectra of the Pellecchiella jam showing the proportion (percentage of surface) of each unique color measured in a 4096-color space if greater than 1%.

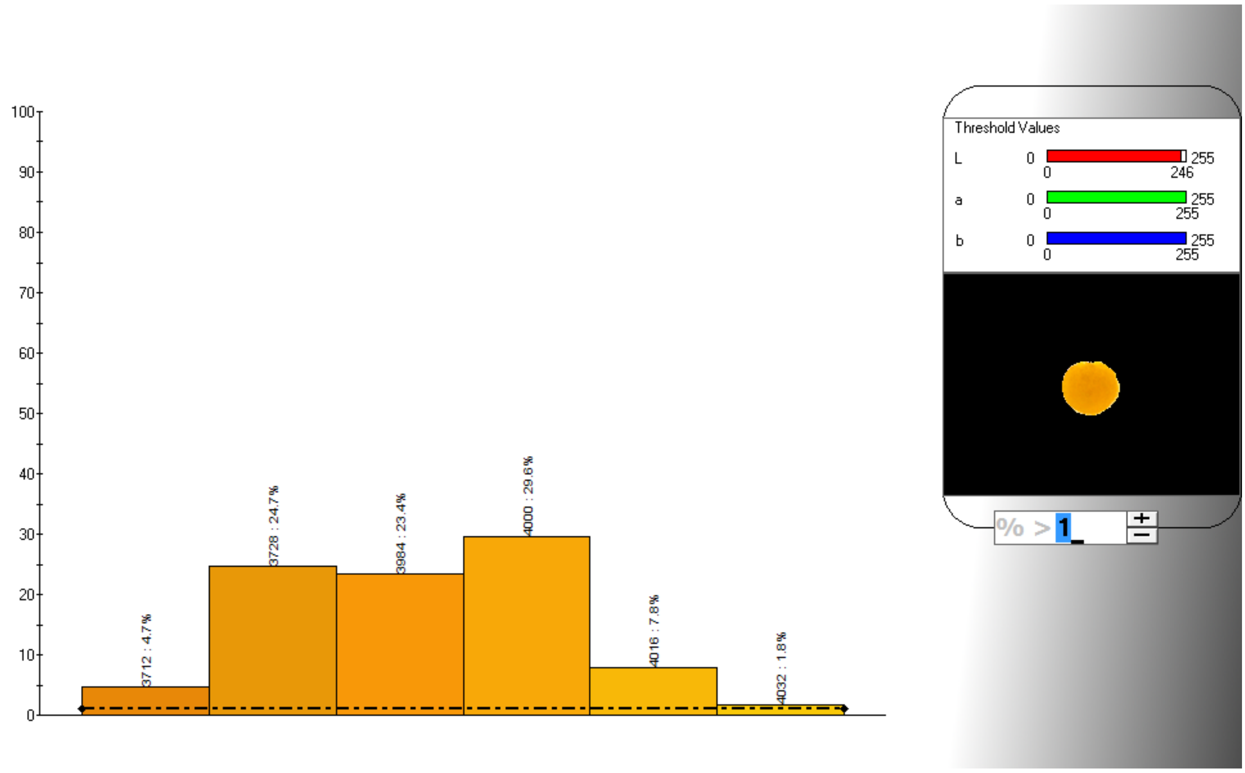

Supplement: Supplementary file 1 [file foods-14-00945-s001.zip › foods-3500222-supplementary.pdf]
